# Supplementary material for: Resolving the full spectrum of human genome variation using Linked-Reads
Source: Genome Res. 2019 Apr;29(4):635–45. doi: 10.1101/gr.234443.118 (PMC6442396; doi:10.1101/gr.234443.118)
Supplement: Supplemental Material [file supp_gr.234443.118_Supplemental_longranger-2.2.zip › longranger-2.2.tar.bz2!/lr2.2-public/lib/stan/cmdstan/stan/doc/api/html/functions_vars_i.html]

Stan: Class Members - Variables


|  |  |
| --- | --- |
|  | Stan  2.5.0  probability, sampling & optimization |


- Main Page
- Related Pages
- Namespaces
- Classes
- Files

- Class List
- Class Index
- Class Hierarchy
- Class Members

- All
- Functions
- Variables
- Typedefs
- Enumerator
- Related Functions

- \_
- a
- b
- c
- d
- e
- f
- g
- h
- i
- k
- l
- m
- n
- o
- p
- q
- r
- s
- t
- u
- v
- w
- x
- y
- z


All Classes Namespaces Files Functions Variables Typedefs Enumerations Enumerator Friends Macros Pages

### - i -

- identifier\_name\_r
  : stan::gm::var\_decls\_grammar< Iterator >
- identifier\_r
  : stan::gm::functions\_grammar< Iterator >
  , stan::gm::statement\_grammar< Iterator >
  , stan::gm::term\_grammar< Iterator >
  , stan::gm::var\_decls\_grammar< Iterator >
- include\_sampling\_
  : stan::gm::statement\_visgen
- increment\_log\_prob\_statement\_r
  : stan::gm::statement\_grammar< Iterator >
- indent\_
  : stan::gm::generate\_init\_vars\_visgen
  , stan::gm::generate\_local\_var\_init\_nan\_visgen
  , stan::gm::statement\_visgen
- indent\_width
  : stan::gm::argument
- indents\_
  : stan::gm::local\_var\_decl\_visgen
  , stan::gm::member\_var\_decl\_visgen
  , stan::gm::validate\_transformed\_params\_visgen
  , stan::gm::validate\_var\_decl\_visgen
- indexed\_factor\_r
  : stan::gm::term\_grammar< Iterator >
- init
  : stan::io::stan\_csv\_metadata
- int\_decl\_r
  : stan::gm::var\_decls\_grammar< Iterator >
- int\_literal\_r
  : stan::gm::term\_grammar< Iterator >
- integrate\_ode\_r
  : stan::gm::term\_grammar< Iterator >
- integrator\_
  : stan::mcmc::base\_hmc< M, P, H, I, BaseRNG >
- is\_bounded
  : std::numeric\_limits< stan::agrad::fvar< T > >
  , std::numeric\_limits< stan::agrad::var >
- is\_exact
  : std::numeric\_limits< stan::agrad::fvar< T > >
  , std::numeric\_limits< stan::agrad::var >
- is\_fun\_return\_
  : stan::gm::generate\_local\_var\_init\_nan\_visgen
  , stan::gm::local\_var\_decl\_visgen
  , stan::gm::statement\_visgen
- is\_iec559
  : std::numeric\_limits< stan::agrad::fvar< T > >
  , std::numeric\_limits< stan::agrad::var >
- is\_integer
  : std::numeric\_limits< stan::agrad::fvar< T > >
  , std::numeric\_limits< stan::agrad::var >
- is\_modulo
  : std::numeric\_limits< stan::agrad::fvar< T > >
  , std::numeric\_limits< stan::agrad::var >
- is\_signed
  : std::numeric\_limits< stan::agrad::fvar< T > >
  , std::numeric\_limits< stan::agrad::var >
- is\_specialized
  : std::numeric\_limits< stan::agrad::fvar< T > >
  , std::numeric\_limits< stan::agrad::var >
- is\_var\_
  : stan::gm::generate\_local\_var\_init\_nan\_visgen
  , stan::gm::init\_local\_var\_visgen
  , stan::gm::local\_var\_decl\_visgen
  , stan::gm::statement\_visgen


---

[ Stan Home Page ]

*© 2011–2014,
Stan Development Team.*
